# Effects of Two Dietary Fibers as Part of Ready-to-Eat Cereal (RTEC) Breakfasts on Perceived Appetite and Gut Hormones in Overweight Women

**DOI:** 10.3390/nu7021245

**Published:** 2015-02-13

**Authors:** David W. Lafond, Kathryn A. Greaves, Kevin C. Maki, Heather J. Leidy, Dale R. Romsos

**Affiliations:** 1 Kellogg Company, WKKI 2 Hamblin Ave E, Battle Creek, MI 49017, USA; E-Mail: kathy.greaves@kellogg.com; 2 Department of Food Science and Human Nutrition, Michigan State University, 469 Wilson Road, East Lansing, MI 48824-1224, USA; E-Mail: dromsos@msu.edu; 3 Biofortis Clinical Research/Midwest Center for Metabolic & Cardiovascular Research, 489 Taft Avenue, Glen Ellyn, IL 60137, USA; E-Mail: kmaki@mc-mcr.com; 4 Department of Nutrition & Exercise Physiology, University of Missouri, 307 Gwynn Hall, Columbia, MO 65211, USA; E-Mail: leidyh@health.missouri.edu

**Keywords:** ready to eat cereal (RTEC), satiety, PYY, GLP-1, arabinoxylans, visual analogue scales (VAS)

## Abstract

The effects of an enzyme-hydrolyzed arabinoxylan from wheat (AXOS) *versus* an intact arabinoxylan from flax (FLAX) added to a ready-to-eat cereal (RTEC) on the postprandial appetitive, hormonal, and metabolic responses in overweight women (BMI 25.0–29.9 kg/m^2^) were evaluated. Subsequent meal energy intake was also assessed. Two randomized, double-blind, crossover design studies were completed. For trial 1, the participants consumed the following RTEC breakfast, matched for total weight and varied in energy content: low-fiber (LF, 4 g); high-fiber (HF, 15 g) as either AXOS or FLAX. For trial 2, the participants consumed LF, HF-AXOS, and HF-FLAX RTECs but also consumed another LF breakfast that was isocaloric (LF-iso) to that of the HF breakfasts. Perceived appetite and blood samples (trial 2 only) were assessed before and after breakfast. An *ad libitum* lunch was offered 4 h post-breakfast. No differences in postprandial appetite responses were observed among any breakfasts in either trial. The HF-AXOS and HF-FLAX led to increased postprandial GLP-1 and peptide YY (PYY) concentrations *vs.* LF-iso. No differences were observed in lunch meal energy intake among breakfast meals in either trial. Collectively, these data suggest that 15 g of low molecular weight fiber added to RTECs did not affect perceived appetite or subsequent energy intake despite differences in satiety hormone signaling in overweight females.

## 1. Introduction

Over the last 25 years there have been many studies examining the role of increased dietary fiber intake on satiety and weight loss. A link between fiber and satiety was proposed as early as 1987 [1]. Since that time there has been a significant amount of research relating fiber to satiety [2,3]. Dosages of fiber used have varied considerably, with more consistent satiety effects above 10 g at a single eating occasion. However, mean daily fiber intake for adult women in the U.S. is only slightly above this threshold (about 14 g/day) [4] and well below the recommended 25 g/day [5].

It is difficult to incorporate fiber sources into existing breakfast cereal food products without changing desirable food texture and flavor characteristics. Fiber may be added either as part of an ingredient, or as an isolated fiber [6]. When fiber is added to food as part of an ingredient such as wheat bran, other components in that ingredient can contribute color, flavor, and texture, as well as additional calories to the finished food. In the case of wheat bran, these components would include starch and protein from the endosperm included in the bran mill fraction. An isolated fiber would contribute significantly fewer calories to the cereal, and less change to food properties.

Addition of insoluble fibers to a breakfast cereal will increase the amount of water needed to process it, water that ultimately must be removed, adding process complexity and costs. Viscous soluble fibers added to cereals may increase the viscosity of the dough stage, making it harder to process through the unit operations needed to make the cereal (extrusion, mixing, pumping, drying) [6]. One approach to reducing the viscosity of soluble fibers is to hydrolyze them into shorter polymers, which then avoids these processing issues.

Novel concentrated fibers are being created by reducing their molecular weights through enzymatic hydrolysis, or by chemical modifications of existing fibers, to facilitate their addition to food, as well as to minimize effects on the finished product [7,8]. Overweight women who are conscious of their body shape and desire to lose weight are seeking food solutions. To help these consumers, the food industry is customizing foods that are lower in calories and higher in fiber content, many containing novel fibers [9,10]. Viscous high molecular weight soluble fibers added to beverages have been shown to increase satiety [11]. Although they have optimized food quality characteristics, it is not certain whether lower molecular weight modified fibers will still have beneficial effects on satiety.

Two randomized, double-blind, crossover design studies were completed to evaluate the effects of an enzyme-hydrolyzed arabinoxylan from wheat (AXOS) *versus* an intact arabinoxylan from flax (FLAX) added to a ready-to-eat cereal on the postprandial appetitive, hormonal, and/or metabolic responses in overweight women. Subsequent meal energy intake was also assessed.

## 2. Materials and Methods

### 2.1. Subjects

For both trials, subjects were overweight women, 18–29 years of age, each with a body mass index (BMI) of 25.0–29.9 kg/m^2^, inclusive. Exclusion criteria included recent weight loss of >4.1 kg within 4 weeks of screening visit; history or presence of cancer, renal, hepatic, endocrine (including diabetes mellitus), pulmonary, biliary, gastrointestinal, pancreatic, or neurologic disorders; recent use of any weight loss drugs; weight-reducing surgery or a diagnosed eating disorder; and pregnancy or planning to become pregnant during the study period. Subjects were regular consumers of breakfast cereal and did not dislike macaroni and cheese.

Subjects were recruited through approved advertisements, flyers, and emails. Before starting the trial, all applicants completed an assessment for inclusion in the study. This included body weight, vital signs, evaluations of inclusion/exclusion criteria, concomitant medication use, a first day of last menses query, as well as medical history, Eating Habits Questionnaire (to exclude unusual eating patterns), in-clinic urine pregnancy test, and Vein Access Scale assessments. Signed written informed consent for participation in the study was obtained from all subjects before protocol-specific procedures were carried out. Subjects were informed of their right to withdraw from the study at any time. These trials were conducted according to Good Clinical Practice Guidelines, the Declaration of Helsinki (2000), and US 21 CFR. The trials were approved by Quorum Review IRB (25787/1 and 26950/1), an appropriately constituted Institutional Review Board, in accordance with the requirements of 21 CFR 56. For trial 1 and 2, 30 and 36 subjects, respectively, were recruited and completed the studies. Five subjects participated in both trials. Women were only tested during the luteal phase of their menstrual cycles (defined as ≥day 15, where day 1 = first day of menses) in order to improve the sensitivity of satiety testing [12,13]. Therefore, before starting each treatment visit, subjects again underwent assessments of body weight, vital signs, evaluation of inclusion/exclusion criteria, concomitant medication use, and a first day of last menses query. For trial 1, treatment visits were a minimum of 7 days apart and for trial 2, visits were a minimum of 4 days apart to enable more treatment visits within the luteal phase of the menstrual cycle and because 4 days was considered to be a sufficient period for washout of any carryover from the previous treatment.

#### 2.1.1. Diets

Wheat bran is a major component of some high-fiber breakfast cereals, with arabinoxylans being the predominant fiber in wheat bran [14]. The hydrolyzed fiber included in this study was a wheat bran arabinoxylan extract (AXOS), enzyme hydrolyzed and purified by a proprietary process and obtained from Fugeia N.V. (Belgium). Several treatment steps designed to remove the digestible carbohydrates, cellulose, and proteins from the bran yielded a concentrated wheat arabinoxylan [8] with a degree of polymerization between 3 and 9. The percent fiber in the hydrolyzed AXOS was determined by AOAC method 2009.01 and was 79.6% [15].

Since an isolated unhydrolyzed wheat bran arabinoxylan was not available in the quantities needed for this work, a flax seed extract composed of arabinoxylans and rhamnogalacturonan was used. The intact fiber (FLAX) was a flax fiber extract obtained from Biogin Biochemicals Co. Ltd. (China). The FLAX was extracted from flax seed mucilage using water and ethanol. The percent fiber in the FLAX was determined by AOAC method 2009.01 and was 81.3% [15].

#### 2.1.2. RTEC, Flaked

RTEC was produced by a proprietary process in the Kellogg Company pilot plant. The main unit operations were cooking under pressure, then cooling and drying the dough, forming pellets, drying and tempering, and finally flaking and toasting. Cooking was done in a batch pressure cooker to heat food to a temperature of 123 °C and 117 kPa pressure to hydrate the grains. The nutrient composition of the cereals is shown in Table 1 and the formulae are referenced [15]. The RTECs were not fortified and any micronutrient losses should be the same in all 4 RTECs.

**Table 1:** Nutrient composition of RTE cereals.

RTE Cereals	LF	LF-iso	HF-AXOS	HF-FLAX	
Flaked Cereal (g)	100	70	100	100	
Water (g)	220	260	200	200	
2% Milk (g)	180	170	200	200	
Mass (g)	500	500	500	500	
Total Carbohydrate (g)	95	69	82	82	
Fiber (g)	4	3	19	19	
Protein (g)	13	11	12	12	
Lipid (g)	5	4	5	5	
Calories (kcal)	461	345	345	347	

LF: low-fiber RTEC; HF-AXOS: high-fiber (HF) RTEC with AXOS fiber; HF-FLAX: high-fiber (HF) RTEC with FLAX fiber; LF-iso: isocaloric RTEC to that of the HF breakfasts.

In trial 1, subjects consumed 100 g of one of the following RTECs with 2% milk on three separate days in random order: low fiber (LF, 4 g); high fiber (HF, 15 g) as AXOS; or high fiber (HF, 15 g) as FLAX. In trial 2, subjects randomly consumed the LF, HF-AXOS, or HF-FLAX RTECs as in trial 1, but also consumed a LF RTEC that was isocaloric to the HF meals (LF-iso); thus, the LF-iso was only 70 g. In both trials, the milk was diluted with water to standardize the weight consumed (500 g/breakfast meal).

#### 2.1.3. Cereal Flake Texture and Sensory Measurements

RTEC (59 mL) was placed in a Kramer shear press and peak force (g) was assessed using TX.XT Plus Texture Analyzer (Stable Micro Systems, Surrey, UK) with 50 kg load cell at a test speed of 2 mm/s. For milk soaked samples, RTE cereal (59 mL) was placed into 52 mL of 2% milk for 7 min. The soaked sample was assessed at a speed of 5 mm/s with a square probe. A trained panel of 8 people also evaluated the three RTEC samples for texture attributes in a sequential monadic fashion both at first bite and after chewing (<15 times). The data were collected by panel consensus [16].

### 2.2. Trial Design

Trials 1 and 2 were both randomized, double-blind, placebo-controlled, crossover trials. In both trials, subjects fasted 9–13 h before the start of RTEC product consumption (*t* = 0 min). Subjects recorded all foods and beverages consumed after 1400 h the day prior to the first treatment visit. These records were collected and reviewed, and subjects were provided a copy of the record and instructed to replicate the same diet from 1400 h the day prior to each subsequent treatment visit. Following the overnight fast, the subjects randomly consumed one of the breakfasts on each testing day between 0630 h and 0930 h. Perceived appetite and satiety were assessed before and after breakfast at specific times throughout the 4.5 h testing day. After the 120 min rating, subjects were allowed 200 mL of water. The amount of water consumed was recorded, and subjects were asked to consume a similar amount of water at subsequent treatment visits.

In addition to the previous procedures, trial 2 also included blood sample collection. An intravenous catheter was inserted in the forearm for collection of venous blood. To maintain patency of the intravenous catheter, the catheter was flushed with 10 mL normal saline solution hourly. A baseline blood sample to assess active ghrelin (acylated form; *n*-octanoic acid on serine 3), active glucagon-like peptide-1 (GLP-1_7–36_), total PYY (PYY_3–36_ + PYY_1–36_), glucose, and insulin was drawn at *t* = −10 ± 5 min. Subsequent blood samples were collected at *t* = 15, 30, 45, 60, 90, 120, 180, and 240 ± 5 min.

### 2.3. Ad Libitum Lunch

A macaroni and cheese lunch (40 oz.) was provided in coded ceramic pots to subjects at 240 min after the breakfast meal (Stouffer’s Family Size 40 oz., Nestlé USA Inc., Solon, OH, USA). Subjects ate directly from the pots and were provided with a standard amount of water during lunch. The quantity of water consumed was recorded. Subjects were allowed 25 min for lunch and were instructed to eat until comfortably full. Food was weighed prior to and following consumption, and energy intake was assessed based on nutrition facts panel data (Total Fat 17.0 g, Carbohydrates 30.0 g, Dietary Fiber 2.0 g, Sugars 4.0 g, Protein 15.0 g, 330 kcal/225 g).

### 2.4. Appetite Measurements

Perceived appetite was assessed using a visual analogue scale (VAS) [17]. The scale was 100 mm in length with descriptions expressing the most positive and most negative ratings for desire to eat, hunger, fullness, and prospective consumption. Subjects placed a mark crossing the line connecting descriptors and the distance in mm from the left side was used as the rating. Perceived appetite was assessed prior to breakfast meal consumption (*t* = −15 and −5 min). At *t* = 0 min, subjects consumed one of the RTECs between 0700 and 1000 h. Subjects were allowed 15 min to consume the breakfast meal. Subsequent VAS ratings were completed by the subjects at *t* = 15, 30, 45, 60, 75, 90, 105, 120, 135, 150, 165, 180, 195, 210, 225, 240, and 270 min. Net incremental area under the curve (niAUC) was calculated using the trapezoid method for 2 h and 4 h.

### 2.5. Hormonal Analyses

Blood was collected in test tubes containing EDTA and protease inhibitors (AEBSF and DPP-IV) to reduce protein degradation. Within 10 min of collection, samples were centrifuged at 4 °C for 10 min. The plasma was separated and stored at −80 °C for future analysis. Plasma active ghrelin (acylated form; n-octanoic acid on serine 3), active GLP-1 (GLP-1_7–36_), total PYY (PYY_3–36_ + PYY_1–36_), and insulin, were prepared using the Milliplex MAP magnetic bead-based multi-analyte, metabolic panel, 4-plex immunoassay (HMHMAG-34K; Millipore, St. Charles, MO, USA) and measured using Luminex Magpix with xPONENT software (Luminex Corporation, Austin, TX, USA). Intra- and inter-assay CV were 2% and 8%, 7% and 10%, 2% and 11%, and 3% and 6% for active ghrelin (acylated form), active GLP-1 (GLP-1_7–36_), total PYY (PYY_3–36_ + PYY_1–36_), and insulin, respectively. The detection limit of the assay was 2, 7, 8, and 58 pg/mL for active ghrelin (acylated form), active GLP-1 (GLP-1_7–36_), total PYY (PYY_3–36_ + PYY_1–36_), and insulin, respectively. Plasma glucose was measured with a glucose oxidase assay using a BioTEk ELx808 Absorbance Microplate Reader (BioTek, Winooski, VT, USA) and Glucose Oxidase Reagent (TR-15221; ThermoFisher Scientific, Austin, TX, USA). Intra- and inter-assay CV for glucose were 2% and 5%. The detection limit of the assay was 1.0 mg/dL.

### 2.6. Gastrointestinal Tolerability

Assessment was by self-reported scoring on a 6-point scale with 0 being not experienced, and 5 being most severe experience for each attribute [18]. Five different gastrointestinal symptoms were measured (cramping, flatulence, gas/bloating, loose stools, and nausea).

### 2.7. Statistics

#### 2.7.1. VAS Appetite Scores

All values are reported as means with their respective standard errors. Statistical analyses were conducted using SAS for Windows (version 9.1.3 or higher, Cary, NC, USA). Descriptive statistics are presented for the outcome parameters for each RTE cereal. Response differences among RTE cereals were assessed using repeated measures analysis of variance (ANOVA) including subject as a random variable, and test diet as a fixed effect. The model was reduced until only test diet and any significant (*p* < 0.05) terms remained. Pairwise comparisons between all treatment conditions were conducted using Tukey’s adjustment for multiple comparisons.

All tests of significance, unless otherwise stated, were performed at α = 0.05, two-sided. Assumption of normality of residuals from the final model of each outcome parameter was investigated by the Shapiro–Wilk test [19].

#### 2.7.2. Hormones and Glucose

All values are reported as means with their respective standard errors. Statistical analyses were conducted using PROC MIXED in SAS for Windows (version 9.2, Cary, NC, USA). Repeated measures over time were modeled with a heterogeneous banded (Toeplitz) covariance structure using the repeated statement of SAS. For all effects, significance was declared at *p* < 0.05. Descriptive statistics are presented for the outcome parameters for each test diet using least squares means. Response differences among breakfast meals were assessed for each time point using mixed model analysis including subject as a random variable, and treatment as a fixed effect. The model was reduced until only test diet and any significant (*p* < 0.05) terms remain. Pairwise comparisons between all treatment conditions were conducted using Tukey’s adjustment for multiple comparisons. Net incremental area under the curve (niAUC) was calculated using the trapezoid method and results reported at 2 and 4 h [20]. Per protocol, hormone analysis values were screened to reject subjects with at least 1 plasma hormone niAUC value greater than 3 times the standard deviation from the mean in at least 2 breakfast meals. One subject did not complete one of the study treatment testing days and thus had no hormonal or glucose data, and one subject was excluded from the glucose data analyses since her fasting plasma glucose concentration was greater than 100 mg/dL. Lastly, three subjects displayed GLP-1 concentrations that were below the assay limit of detection. Thus, the number of data points per hormone and per breakfast meal varied.

#### 2.7.3. Sample Size

In trial 1, with an evaluable sample of 30 subjects, the study was projected to have 80% power to detect a difference of 10 mm in the appetite score (pre-meal to 240 min) between treatments assuming a standard deviation of 15.9 mm based on previous work completed by the investigators. This assumes a nominal *p*-value of 0.017 to account for up to three primary comparisons using a Šidák adjustment [21]. Rolling recruitment was employed until the desired 30 subjects had been randomized. In trial 2, with an evaluable sample of 33 subjects, the study was projected to have 80% power to detect a difference of 9.8 mm in the average appetite composite score (pre-meal to 240 min) between treatments assuming a standard deviation of 15.9 mm. This assumes a nominal *p*-value of 0.0127 to account for up to four primary comparisons using a Šidák adjustment. A sample of 36 subjects was randomized to allow for attrition and non-compliance. After consuming control A RTE cereal, 27 subjects were included for GLP-1, and 30 subjects for the remaining hormone assays and glucose. After consuming the remaining breakfast RTE cereals, 28 subjects were included for GLP-1, and 31 subjects for the remaining hormone assays and glucose.

## 3. Results

### 3.1. Cereal Flake Texture and Sensory Characteristics

The composition of RTECs was similar in protein, fat (Table 1), and flake density (133, 136, and 166 kg/m^3^ for the LF, HF-AXOS, and HF-FLAX RTECs, respectively) to minimize the impact of these factors in the comparisons. RTEC texture was evaluated by both instrumental and sensory methods. The 3 RTECs had similar texture based on peak force measurements both dry and in milk (dry: 9744 ± 1158, 7882 ± 722, and 7369 ± 1288 g of force, and in milk: 35,588 ± 7305, 35,506 ± 8196, and 30,667 ± 3598 g of force for LF, HF-AXOS and HF-FLAX RTECs, respectively). There were slight texture differences in sensory evaluation of the 3 RTE cereals (Table 2). The LF flake exhibited a more cohesive mass after chewing and in milk. The HF-AXOS RTEC absorbed the least moisture/saliva after chewing. Finally, the HF-FLAX RTEC was the least gritty. Flavor was also judged to be very similar (data not shown).

**Table 2:** Sensory characteristics of RTE flaked cereal dry and with milk. Results were collected as panel consensus ^a^.

Attribute Title	LF	HF-AXOS	HF-FLAX
First Chew (Dry Cereal)			
Hardness, Molars	5.0	5.5	5.0
Fracturability	4.5	5.0	4.0
Chew Down (Dry Cereal)			
Moisture Absorption	11.0	7.5	10.0
Cohesiveness of Mass	8.5	7.5	8.0
Gritty/Particles	4.0	3.0	2.0
3 Min in Milk			
Fracturability	0.0	0.0	0.0
Cohesiveness of Mass	5.5	4.5	4.0

^a^ 15-point scale divided into ½ point increments, with 0 meaning “none” and 15 meaning “extremely strong” [16] LF: low-fiber RTEC; HF-AXOS: high-fiber (HF) RTEC with AXOS fiber; HF-FLAX: high-fiber (HF) RTEC with FLAX fiber.

### 3.2. Subjects

Subject characteristics for each trial are outlined in Table 3. Characteristics of the participants were controlled with inclusion of females within a narrow range of BMI and age. Since the high fiber diets contained 19 g of fiber per serving (15 g test fiber and 4 g base cereal fiber), gastrointestinal tolerability was assessed in both trials (data not presented for trial 1) at each treatment [18].

**Table 3:** Subject characteristics at baseline.

Parameter	Trial 1	Trial 2
Female	30	36
Race/ethnicity		
Non-Hispanic White	20	23
Black/African American	8	8
Asian or Pacific Islander	1	2
Multiracial	1	3
Smoking Status		
Non-Smoker	24	27
Current Smoker	2	5
Past Smoker	4	4
Age (years)	22.5 (0.6) ^a^	24.3 (0.5)
Weight (kg)	72.9 (1.2)	74.3 (1.2)
Body Mass Index (kg/m^2^)	27.0 (0.3)	27.4 (0.3)
Systolic Blood Pressure (mm Hg)	109 (2)	113 (2)
Diastolic Blood Pressure (mm Hg)	66 (1)	71 (2)
Heart Rate (bpm)	73 (2)	77 (2)

^a^ standard error of mean in parentheses.

Gastrointestinal symptoms were not observed in a majority (66%–75%) of the participants. However, for those who did experience symptoms during the study, flatulence was higher with the high-fiber containing RTECs (1.3 ± 0.3 and 1.1 ± 0.3 for HF-AXOS and HF-FLAX RTECs, respectively) compared with both control diets (0.3 ± 0.2 and 0.2 ± 0.1 for LF and LF-iso RTECs, respectively). Gas/bloating experienced was also higher with the high-fiber containing RTECs (1.5 ± 0.3 and 1.4 ± 0.3 for HF-AXOS and HF-FLAX RTECs, respectively) compared with both LF controls (0.3 ± 0.2 and 0.1 ± 0.1 for LF and LF-iso RTECs, respectively).

### 3.3. Trial 1

**Figure 1:**
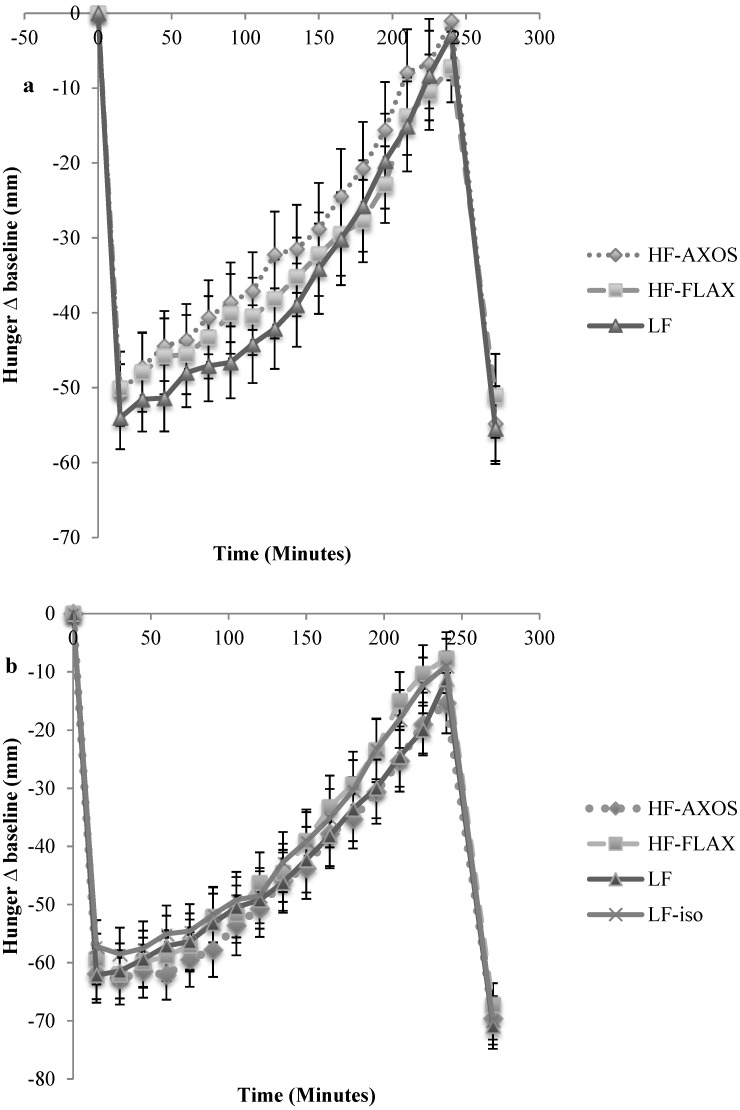
Hunger Score change from baseline following consumption of each breakfast. (**a**) Clinical Trial 1. Data are presented as mean ± SEM, based on 30 subjects. (**b**) Clinical Trial 2. Data are presented as mean ± SEM, based on 36 subjects. There were no significant differences between treatments at any time point (*p* > 0.05) in either trial. LF: low-fiber RTEC; HF-AXOS: high-fiber (HF) RTEC with AXOS fiber; HF-FLAX: high-fiber (HF) RTEC with FLAX fiber; LF-iso: isocaloric RTEC to that of the HF breakfasts.

Increasing fiber content from 4 g/serving in the control cereal to 19 g/serving in the two high-fiber cereals did not affect perceived appetite ratings. Results for VAS scores (mm) of Hunger, Fullness, Desire to Eat, and Prospective Consumption showed no significant differences (*p* > 0.05) among treatment conditions in appetite ratings at any time point (only Hunger data presented, Figure 1). Total niAUC_0–240_ values for Hunger over the pre *ad libitum* lunch phase were −8379 ± 1159, −7064 ± 1211, and −7928 ± 1234 mm × minutes ± SEM for the control, AXOS, and FLAX RTE cereals, respectively. No significant differences were observed among conditions.

The energy intake subjects consumed during the *ad libitum* lunch did not differ significantly among treatments (*p* = 0.96), and thus subjects did not compensate for lower calorie intakes with the high-fiber breakfasts (breakfast calories 461, 345, 347 kcal for LF, HF-AXOS and HF-FLAX RTECs, respectively). Mean energy intakes during the *ad libitum* lunch were 552 ± 37, 545 ± 37, and 559 ± 36 kcal ± SEM for the LF, HF-AXOS, and HF-FLAX RTE cereals, respectively. Mean total energy intakes from the combined RTEC breakfast and lunch meals were 1013 ± 36, 907 ± 37, and 906 ± 36 kcal ± SEM for the LF, HF-AXOS, and HF-FLAX RTECs, respectively (*p* = 0.065 across breakfasts). Consequently participants consumed ~10% fewer kcal for breakfast and lunch combined when fed the HF-AXOS (*p* = 0.109) and HF-FLAX (*p* = 0.101) RTECs *versus* the LF RTEC because the LF RTEC breakfast contained more calories. When both fiber treatments were averaged together, the difference in energy intake reached significance *versus* control (*p* = 0.036).

### 3.4. Trial 2

In this trial, 3 of the diets were the same as in trial 1. An additional control was added to compare a low-fiber control RTEC with high calories (LF; 4 g fiber; 461 kcal), to a low-fiber control RTEC (LF-iso; 3 g fiber; 341 kcal) energy matched to the high-fiber RTECs. As with trial 1, increasing fiber content from 3 to 4 g/serving in the two control cereals to 19 g/serving in the two high-fiber cereals did not affect appetite ratings. Results for VAS scores of Hunger, Fullness, Desire to Eat, and Prospective Consumption showed no significant differences (*p* > 0.05) among treatment conditions in appetite ratings at any time point (only Hunger data presented, Figure 1). Total niAUC_0–240_ values for Hunger over the pre *ad libitum* lunch phase were −10,342 ± 1054, −9566 ± 1037, −10,723 ± 1060, −9691 ± 1046 mm × minutes ± SEM for the LF, LF-iso, HF-AXOS, and HF-FLAX RTECs, respectively. No significant differences were observed among RTECs.

As in trial 1, subjects consumed similar amounts of macaroni and cheese during the *ad libitum* lunch in the four treatment conditions. Again, just as in the first clinical trial, they did not compensate for the lower calories in the isocaloric breakfasts (461, 341, 345, 347 kcal for the LF, LF-iso, HF-AXOS and HF-FLAX RTECs, respectively). Energy intakes from lunch were 533 ± 37, 558 ± 38, 544 ± 37, and 547 ± 36 kcal (mean ± SEM) for the LF, LF-iso, HF-AXOS, and HF-FLAX RTECs, respectively and were not significantly different between treatments (*p* = 0.77). Total energy intakes from the combined RTEC and lunch for the LF-iso 899 ± 38, HF-AXOS 907 ± 37, and HF-FLAX 894 ± 36 kcal (mean ± SEM) conditions were significantly lower than for LF, 994 ± 37 kcal (*p* ≤ 0.006 for each), again suggesting that the participants did not compensate for the lower energy intake at the breakfast meal.

Since the 2 high-fiber cereals and the high-energy control cereal were the same in both clinical trials, the niAUC data were pooled for these test conditions. Although this served to increase the number of subjects included in the analysis (*n* = 65), there was still no significant difference in appetite among these test cereals (*p* > 0.05) at any time point.

### 3.5. Hormones and Glucose

Blood samples were collected from each of the subjects after the overnight fast prior to each day they ate the 4 different breakfasts. Five subjects were removed from the data pool per protocol. One subject was removed due to high fasting plasma glucose concentrations (>200 mg/dL) on all four days. The other 4 subjects were removed because one or more of their niAUC hormone concentrations were >3 standard deviations from the mean for at least 2 breakfast meals. Baseline values for each of the hormones and glucose, after these subjects were removed, were similar prior to consumption of the 4 breakfasts. Fasted average glucose concentrations were in the normal range, consistent with screening criteria for the study to exclude subjects that had diabetes or pre-diabetes.

#### 3.5.1. Ghrelin

**Figure 2:**
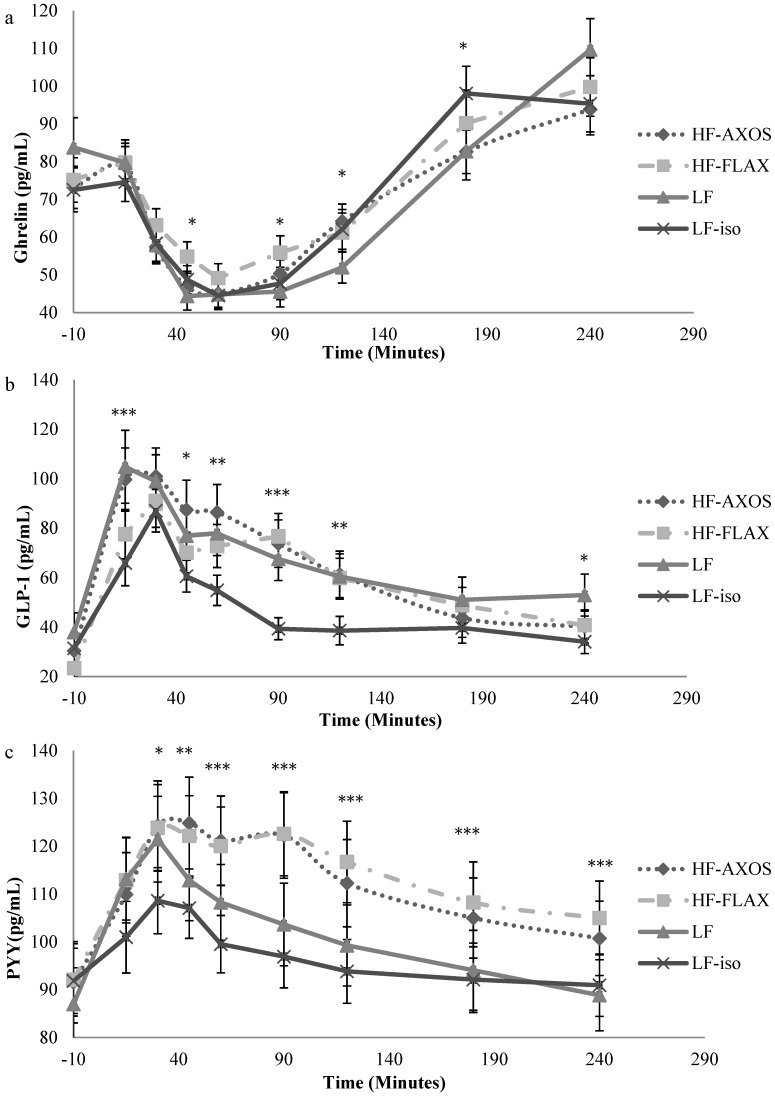
Plasma hormone concentrations at selected time points: (**a**) active ghrelin, (**b**) active GLP-1, (**c**) total PYY. Significant differences among groups are signified as follows: *** *p* < 0.001; ** *p* < 0.01; * *p* < 0.05. LF: low-fiber RTEC; HF-AXOS: high-fiber (HF) RTEC with AXOS fiber; HF-FLAX: high-fiber (HF) RTEC with FLAX fiber; LF-iso: isocaloric RTEC to that of the HF breakfasts.

Similar post-meal reductions in plasma ghrelin were observed for all 4 breakfast meals (Figure 2a). Ghrelin concentrations all decreased during the first 45 min. Ghrelin then began to return to baseline values at approximately 60 min and continued to increase until lunch was consumed. The greatest reduction of ghrelin after consumption of breakfast, as well as the longest delay to return to baseline concentrations, tended to occur when subjects consumed the high-energy LF RTEC. A treatment × time interaction was observed with repeated measures, *p* = 0.01. At *t* = 45 and *t* = 90 min, ghrelin concentrations in subjects consuming LF RTEC were significantly lower (*p* < 0.05) than when they consumed HF-FLAX RTEC. At *t* = 120 min, ghrelin concentration measurements for subjects consuming LF RTEC were significantly lower (*p* < 0.05) than when they consumed LF-iso and HF-AXOS RTECs. At *t* = 180 min, ghrelin concentrations in subjects consuming LF RTEC were significantly lower (*p* < 0.05) than when they consumed LF-iso RTEC. Composition of the RTECs did not affect mean niAUC measurements of plasma ghrelin concentrations in subjects after breakfast consumption (Table 4).

**Table 4:** Net incremental area under the curve (niAUC) for each hormone at 2 h and at 4 h: (a) 2 h niAUC, (b) 4 h niAUC.

Blood Parameter *^a^* (mean × 10^3^)	LF	LF-iso	HF-AXOS	HF-FLAX	*p*-Value
(a)					
Ghrelin (pg/mL × min) *^b^*	6.45 (.51) ^A^	6.77 (.51) ^A^	6.67 (.51) ^A^	7.19 (.51) ^A^	0.33
GLP-1 (pg/mL × min)	8.81 (1.11) ^A^	6.27 (1.11) ^B^	9.25 (1.11) ^A^	8.35 (1.11) ^A^	<0.01
Total PYY(pg/mL × min)	12.76 (1.02) ^AB^	11.87 (1.01) ^B^	13.93 (1.02) ^A^	13.93 (1.01) ^A^	<0.01
Glucose (mg/dL × min)	11.59 (.32) ^A^	11.80 (.32) ^A^	11.29 (.32) ^A^	11.18 (.32) ^A^	0.052
Insulin (pg/mL × min)	327.90 (32.62) ^A^	299.80 (32.52) ^AB^	282.80 (32.62) ^B^	269.00(32.52) ^B^	<0.01
(b)					
Ghrelin (pg/mL × min)	11.32 (.88) ^A^	12.06 (.88) ^A^	11.40 (.89) ^A^	12.25 (.88) ^A^	0.33
GLP-1 (pg/mL × min)	12.06 (1.56) ^A^	8.27 (1.56) ^B^	11.96 (1.56) ^A^	11.27 (1.55) ^A^	<0.01
Total PYY(pg/mL × min)	18.41 (1.49) ^BC^	17.40 (1.49) ^C^	20.19 (1.49) ^AB^	20.44 (1.49) ^A^	<0.01
Glucose (mg/dL × min)	16.16 (.37) ^A^	16.41 (.36) ^A^	16.01 (.37) ^A^	15.75 (.36) ^A^	0.12
Insulin (pg/mL × min)	436.00 (42.19) ^A^	382.30 (42.08) ^B^	360.30 (42.19) ^B^	348.70 (42.08) ^B^	<0.01

*^a^* number of subjects varied: LF—GLP-1, 27; Ghrelin, Total PYY, Glucose, Insulin, 30; remaining breakfasts—GLP-1, 28; Ghrelin, Total PYY, Glucose, Insulin, 31; *^b^* standard error of mean in parentheses (±SEM), values within rows with different capital letters represent significant differences using Tukey multiple comparisons and the *p*-value represents treatment differences. LF: low-fiber RTEC; HF-AXOS: high-fiber (HF) RTEC with AXOS fiber; HF-FLAX: high-fiber (HF) RTEC with FLAX fiber; LF-iso: isocaloric RTEC to that of the HF breakfasts.

#### 3.5.2. GLP-1

GLP-1 concentrations immediately increased after consumption of each of the 4 breakfast meals, peaked after 30 min, and slowly decreased towards baseline, remaining above baseline values throughout the remainder of the testing day (Figure 2b). Overall, GLP-1 concentrations tended to be lower when LF-iso RTEC was consumed. When subjects consumed LF-iso RTEC, plasma concentrations of GLP-1 were significantly lower (*p* < 0.05) than when subjects consumed HF-FLAX RTEC at *t* = 90 and 120 min. Concentrations of GLP-1 were lower in subjects consuming LF-iso RTEC than when they consumed LF RTEC at *t* = 15, 90, 120, and 240 min. Concentrations of GLP-1 were also lower in subjects when they consumed LF-iso RTEC than when they consumed HF-AXOS RTEC, with significant differences (*p* < 0.05) in the later portion of the time curve at *t* = 15, 45, 60, 90, and 120 min. Concentrations of GLP-1 were the same in subjects consuming LF RTEC as when subjects consumed either the HF-AXOS or the HF-FLAX RTEC at all time points. Mean niAUC measurements of GLP-1 values demonstrated that subjects consuming LF-iso RTEC had a lower plasma concentration of GLP-1 than when consuming the other 3 treatment cereals at 2 and 4 h (Table 4).

#### 3.5.3. PYY

PYY concentrations immediately increased after consumption of each of the 4 breakfast meals, peaked after 30 min, and then slowly decreased throughout the remainder of the testing day (Figure 2c). A treatment–time interaction was observed with repeated measures, *p* = 0.01. Subjects consuming the two high-fiber RTECs had significantly higher (*p* < 0.05) PYY concentrations than when they consumed the two LF cereals, suggesting a fiber effect on PYY concentrations. Subjects consuming HF-AXOS RTEC also had plasma PYY concentrations higher than when they consumed LF-iso RTEC at almost every time point, with significant differences (*p* < 0.05) at *t* = 30, 45, 60, 90, 120, and 180 min. Overall, PYY concentrations tended to be lowest when subjects consumed LF-iso RTEC. When subjects consumed HF-FLAX RTEC, PYY concentrations were higher than when they consumed LF-iso, with significant differences (*p* < 0.05) at *t* = 30, 45, 60, 90, 120, 180, and 240 min. When subjects consumed LF-iso RTEC, they tended to have lower plasma PYY concentrations than when they consumed the high-energy LF RTEC. Subjects consuming HF-AXOS RTEC had a significantly higher (*p* < 0.05) PYY concentration than when they consumed LF RTEC at *t* = 90. niAUC measurements of PYY concentrations were higher in subjects consuming HF-FLAX RTEC than when they consumed LF-iso RTEC at 2 h, and when they consumed LF-iso or LF RTECs at 4 h (Table 4). Mean niAUC measurements of PYY concentrations were also higher in subjects consuming HF-AXOS RTEC than when they consumed LF-iso RTEC at both 2 and 4 h.

#### 3.5.4. Glucose and Insulin

**Figure 3:**
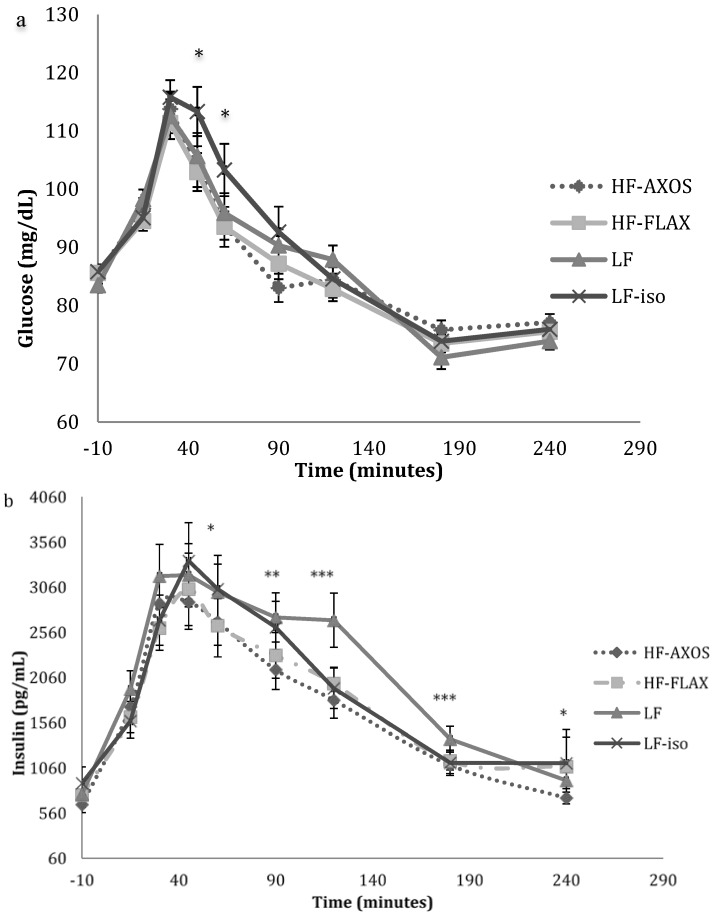
Plasma glucose and insulin concentrations at selected time points: (**a**) glucose, (**b**) insulin. Significant differences among groups are signified as follows: *** *p* < 0.001; ** *p* < 0.01; * *p* < 0.05. LF: low-fiber RTEC; HF-AXOS: high-fiber (HF) RTEC with AXOS; HF-FLAX: high-fiber (HF) RTEC with FLAX fiber; LF-iso: isocaloric RTEC to that of the HF breakfasts.

Glucose concentrations increased immediately after consumption of each of the 4 breakfast meals, reached a peak at 30 min, and then decreased during the remainder of the test (Figure 3a). Subjects’ glucose concentrations all returned to baseline between 120 and 180 min. Plasma glucose concentrations were higher when subjects consumed LF-iso than when they consumed HF-FLAX RTEC, with significant differences (*p* < 0.05) at *t* = 45 and 60 min. Glucose concentrations in subjects consuming all 4 breakfast cereals were not significantly different at the other time points. Mean niAUC measurements of glucose concentrations of subjects consuming all 4 RTECs were not statistically different at 2 or 4 h (Table 4).

Insulin concentrations also increased immediately after consumption of each of the 4 breakfast meals, reaching a peak between 30 and 45 min, and then decreased during the remainder of the test (Figure 3b). Unlike glucose, subjects’ insulin concentrations did not return to baseline until approximately 240 min. Insulin concentrations were higher in subjects consuming the high energy LF RTEC than when they consumed the other breakfasts, with significant differences (*p* < 0.05) *versus* LF-iso RTEC at *t* = 120 and 180 min; *versus* HF-AXOS RTEC at *t* = 90, 120, 180, and 240 min; and *versus* HF-FLAX RTEC at time points *t* = 60, 90, 120, and 180 min. Mean niAUC measurements of insulin concentrations were higher in LF RTEC than HF-AXOS and HF-FLAX RTECs at 2 h and higher than LF-iso, HF-AXOS, and HF-FLAX RTECs at 4 h (Table 4).

## 4. Discussion

The effects of increased dietary fiber on appetite control and satiety are inconsistent, potentially due to type of fiber, dosage, and delivery form incorporated in these studies as well as the physicochemical properties of the fibers [1,2,3]. Also, fibers can be modified by hydrolysis to facilitate incorporation into food, making their effects on satiety even more uncertain. It is important to further understand how the physicochemical properties of fiber and fiber modifications influence satiety and energy intake parameters. This study was designed to evaluate the effects of an enzyme-hydrolyzed arabinoxylan from wheat (AXOS) *versus* an intact arabinoxylan from flax (FLAX) added to a RTEC on perceived appetite, on subsequent meal energy intake, and on hormonal responses in overweight women. No differences in perceived appetite and satiety or subsequent meal energy intake were observed between the low-fiber *versus* the high-fiber breakfast meals in both trials. However, the high fiber breakfast meals led to elevated plasma GLP-1 and PYY concentrations compared to the low-fiber versions. This was an acute study design; longer-term studies are needed to further understand these physicochemical properties of fiber on satiety.

### 4.1. Perceived Appetite

There have been numerous studies comparing perceived appetite after consuming RTECs with added fiber. No effect was found in perceived hunger or fullness (AUC) when comparing RTEC with 41 g fiber, 250 kcal, and a 550 g meal size with a low-fiber control cereal (1.5 g fiber) 60 or 120 min after the breakfast meal [22]. Samra and Anderson [23] provided a RTEC with 33 g fiber and a meal size of 500 g, and found that average appetite scores 75 min later were not statistically different *versus* a low-fiber (1.5 g) RTE cereal. They did observe an increase in fullness with the higher fiber RTEC. Other studies compared RTECs with lower fiber concentrations on perceived appetite [24,25,26,27]. The majority of these studies indicate that there was no effect of fiber on perceived appetite in this food form. The results from the current study support this conclusion. This is in conflict with many studies where a modest amount of fiber added to beverages increased perceived appetite [11]. Food form may have an impact on the ability of fiber to increase satiety.

In a beverage system, as little as 2.5 g fiber has been shown to affect perceived appetite [28,29]. Significant hunger reduction (−13%) with a psyllium (7.4 g) containing beverage relative to no fiber control was found [30]. These studies demonstrated a perceived appetite effect of beverages at much lower added fiber levels than we used in RTECs. One possible explanation is that fibers need to be hydrated before their viscous properties can be fully realized, and this is more likely to occur in a beverage food form than a solid one, or than in a meal with liquid added to a solid form, like milk added to RTEC. In the current study ~80% of the meal mass was liquid (Table 1); however, the physical form of the RTE flake may have attenuated the fiber’s ability to affect perceived appetite.

Viscosity of the fiber is believed to be responsible for some of the satiety effects of beverages [11,29,31]. Kristensen and Jensen [32] reviewed nine published studies on the effect of fiber and viscosity on satiety of beverages. They found that in most cases increased viscosity led to increased satiety with fiber levels tested ranging from 3 to 10 g. Many of these beverage studies used very viscous fibers like alginates and gums that have a very high molecular weight and impact viscosity at a low concentration. The FLAX ingredient we used to produce the high-fiber RTEC had a high molecular weight and a very high viscosity [15]. We anticipated that it would produce a higher viscosity RTEC; however, we found that processing reduced the FLAX fiber molecular weight and viscosity of the cereal. The most viscous cereal at the time of consumption was the LF RTEC (from the higher available gelatinized starch present); however, that viscosity might quickly be reduced by digestive amylases in the intestine. Most RTEC studies used a low-fiber, corn flake control RTEC that had a relatively high level of gelatinized starch. Although viscosity of cereal is seldom reported, high-fiber RTECs may not be more viscous than low-fiber, high-starch control RTECs. This may contribute to the reported inconsistency of high-fiber cereals to enhance satiety.

### 4.2. Lunch Meal Energy Intake

The overweight female subjects in the current study were offered the *ad libitum* lunch meal 4 h after the breakfast meals because this represented a typical time interval between breakfast and lunch meals for most adults. In the current study, subjects consumed a similar amount of calories at lunch regardless of breakfast calories consumed. By 4 h, the effects of the breakfast cereals at the lunch meal may have been diminished, since perceived appetite scores were almost at baseline. In addition, both clinical trials studied overweight female subjects, who may be less responsive to appetite cues [33]. Other investigators also found similar energy intake at lunch offered between 3 and 3.5 h after subjects consumed RTEC with high fiber content [24,25]. However, unlike these studies, some investigators using RTEC did observe a reduction in second meal energy intake when they employed a shorter time interval between breakfast and lunch [23,27,34]. This may explain why they observed a lower energy intake in the second meal.

While we cannot rule out the possibility that the high palatability of the macaroni and cheese lunch meal could also have been a factor biasing toward high energy intake and failure to show a difference between conditions in energy intake despite differences in appetite-related hormones (discussed below), it seems unlikely to have been a material issue influencing the results, given that both VAS and energy intake results were concordant in both trials. It is also possible that the subjects stopped eating due to sensory-specific satiety given they were able to consume only one type of food, contributing to the discrepancy between the hormone and the food intake data [35].

### 4.3. GLP-1 and PYY

Plasma GLP-1 and PYY concentrations were significantly higher with the high-fiber breakfasts than with an equal calorie low-fiber control RTEC. Secretion of GLP-1 and PYY has been shown to be co-located within the enteroendocrine L cells located predominately in the distal small intestine and colon [36], and carbohydrates and protein are strong stimulants of these hormones within this segment of the intestinal tract [37,38,39,40,41]. The high fiber content of the HF-AXOS and HF-FLAX RTECs may have caused carbohydrate and protein residues to reach the distal small intestine within the first hour after consumption [39,42]. Digestion and absorption of carbohydrate and protein in the distal small intestine would then contribute to greater observed elevation of GLP-1 and PYY when the high-fiber RTECs were consumed than when the low-fiber RTECs were consumed.

In the later portion of the interval between breakfast and lunch consumption, it is likely that short-chain fatty acids (SCFA) were produced from the fermentation of the HF-AXOS and HF-FLAX high-fiber containing RTECs, and that the SCFA stimulated L cells in the distal small intestine and colon to cause GLP-1 and PYY secretion [43,44]. Our subjects reported an increase in flatulence and gas/bloating after consuming the high-fiber RTECs, indicating that fermentation was occurring and SCFA were likely being formed [45,46]. The high solubility and low molecular weight of the two fibers added to our high-fiber RTECs likely contributed to rapid fermentation and SCFA production [15,47].

### 4.4. Plasma Ghrelin, Glucose, and Insulin

Plasma ghrelin concentrations were similar after consumption of all 4 breakfast cereals and followed an expected postprandial pattern consistent with our reported VAS appetite scores. Ghrelin is strongly associated with perceived hunger [48], and we observed a time-dependent change in appetite (hunger) and ghrelin concentration. We did observe a lower plasma active ghrelin concentration at the 2 and 3 h time points with the high-energy control RTEC *versus* the low-energy control RTEC, suggesting the greater available calories in the high-energy RTEC might have delayed the return to baseline concentrations in ghrelin. The ghrelin difference observed was relatively small, and may not have been sufficient to elicit a perceived appetite change nor a change in lunch meal energy intake. These lower ghrelin concentrations with the high-energy control RTEC at 2 h and 3 h also corresponded to a higher concentration of insulin at the same time points. Blom *et al.* [49] demonstrated a significant negative correlation between plasma ghrelin and insulin concentrations between 30 and 180 min and concluded that insulin may contribute to ghrelin suppression.

Viscous fiber consumption has been shown to attenuate plasma glucose and insulin concentrations [50,51]. The steam pressure-cooking used to make the RTEC reduced the FLAX fiber molecular weight and viscosity of the cereal such that cereals containing FLAX and AXOS each had a relatively low viscosity [15]. This reduced the FLAX fiber’s ability to attenuate plasma glucose and insulin concentrations.

## 5. Conclusions

No differences in perceived appetite, satiety, or lunch intake were observed with 15 g of added AXOS or FLAX fiber in a RTEC breakfast when consumed by overweight women. Collectively, these data suggest that 15 g of low molecular weight fiber added to RTEC did not affect perceived appetite, satiety, or energy intake at lunch 4 h after breakfast despite differences in satiety hormone signaling in overweight females.
